# Mechanical role of the spatial patterns of contractile cells in invagination of growing epithelial tissue

**DOI:** 10.1111/dgd.12374

**Published:** 2017-07-13

**Authors:** Yasuhiro Inoue, Tadashi Watanabe, Satoru Okuda, Taiji Adachi

**Affiliations:** ^1^ Institute for Frontier Life and Medical Sciences Kyoto University Kyoto 606‐8507 Japan; ^2^ RIKEN Center for Developmental Biology Kobe 650‐0047 Japan

**Keywords:** 3D vertex model, apical constriction, epithelial invagination, multicellular dynamics simulation, spatial pattern

## Abstract

Epithelial invagination is one of the fundamental deformation modes during morphogenesis, and is essential for deriving the three‐dimensional shapes of organs from a flat epithelial sheet. Invagination occurs in an orderly manner according to the spatial pattern of the contractile cells; however, it remains elusive how tissue deformation can be caused by cellular activity in the patterned region. In this study, we investigated the mechanical role of the spatial patterns of the contractile cells in invagination of growing tissue using multicellular dynamics simulations. We found that cell proliferation and apical constriction were responsible for expanding the degree of tissue deformation and determining the location of the deformation, respectively. The direction of invagination depended on the spatial pattern of the contractile cells. Further, comparing the simulation results of surface and line contractions as possible modes of apical constriction, we found that the direction of invagination differed between these two modes even if the spatial pattern was the same. These results indicate that the buckling of the epithelial cell sheet caused by cell proliferation causes the invagination, with the direction and location determined by the configuration of the wedge‐shaped cells given by the spatial pattern of the contractile cells.

## Graphical abstract

The buckling of the epithelial cell sheet caused by cell proliferation causes the epithelial invagination, with the direction and location determined by the configuration of the wedge‐shaped cells given by the spatial pattern of the contractile cells.

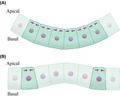

## Introduction

In morphogenesis, deformation of epithelial tissue is essential for the formation of three‐dimensional (3D) epithelial organs. Invagination is a key deformation mode required to fold a planar epithelial sheet into a 3D shape. Epithelial tissue invagination is passively induced by ambient mechanical influences and actively induced by cellular activities such as cell proliferation and apical constriction. Cell proliferation changes the cell volume during cell growth and increases the number of cells by cell division (Mao *et al*. 2011, 2013; Amar & Jia 2013). Elastic energy can be stored in the tissue during cell proliferation, inducing subsequent tissue deformations. Apical constriction affects the deformation of epithelial tissue by causing the cells to actively change from a columnar shape into a wedge‐like shape. In addition, it is known that there are variations in the cellular mechanisms of apical constriction (Martin & Goldstein 2014). Surface contraction occurs when cells accumulate F‐actin on their apical side to form a thick F‐actin band that squeezes their apical surface (Lee & Harland 2007; Sawyer *et al*. 2010; Martin & Goldstein 2014). Line contraction occurs when apical constriction occurs locally on an edge between adjacent cells. Examples of line contraction include supra‐cellular myosin cables (Nishimura *et al*. 2007; Kondo & Hayashi 2015) and planar‐cell‐polarity based machinery (Mao *et al*. 2011; Nishimura *et al*. 2012). It remains to be elucidated how a particular apical constriction mode affects tissue deformation.

In the process of epithelial tissue invagination, cellular activities are known to occur in an orderly manner according to known spatial patterns, such as band‐shaped (Sweeton *et al*. 1991; Smith & Schoenwolf 1997), circular (Simões *et al*. 2006), or circumferential patterns (Nishimura *et al*. 2007). These patterns have been studied by regulating expression of genes controlling cellular activities (Solnica‐Krezel 2005; Fuccillo *et al*. 2006; Suzuki *et al*. 2012; Jidigam *et al*. 2015). In addition to uncovering the molecular mechanism for these patterned cellular activities, there should be a mechanical basis for understanding morphogenesis (Heisenberg & Bellaïche 2013). For instance, mechanics can be used to determine how the spatial pattern controls the force distribution in the tissue, and what kind of tissue deformation results.

In this study, we investigate the mechanical role of the spatial pattern of contractile cells in the epithelial invagination of growing tissue using multicellular dynamics simulations. Further, we examine how a particular apical constriction mode affects epithelial invagination. A 3D vertex model was developed to perform multicellular dynamics simulations (Honda *et al*. 2004; Okuda *et al*. 2013a), and tissue deformations were successfully reproduced based on a force balance at the cellular level (Honda *et al*. 2008a,b; Okuda *et al*. 2013b; Inoue *et al*. 2016). This model can be used to examine mechanical effects of the spatial pattern of cell activity under various conditions on tissue deformation.

## Mathematical model

### 3D vertex model expressing multicellular dynamics

**Figure 1:**
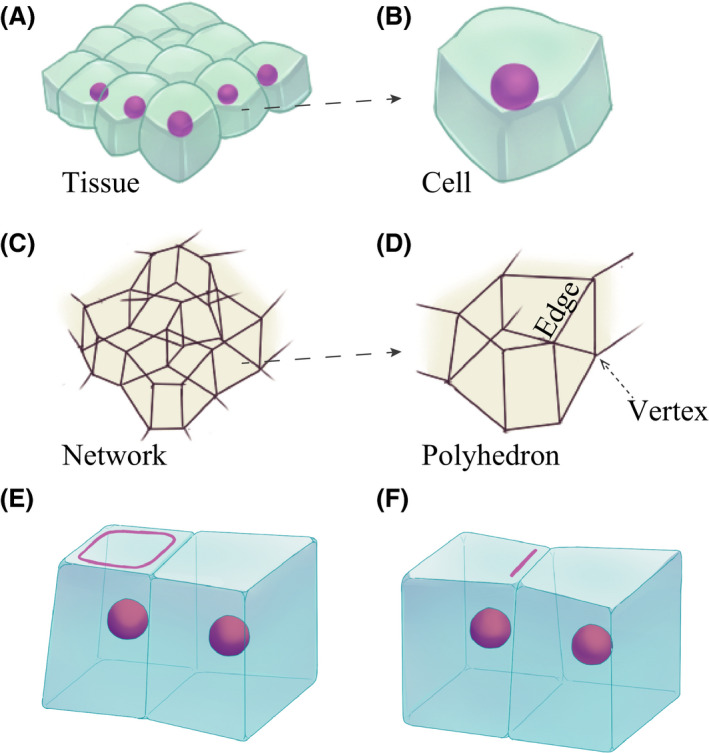
Shapes of (A) epithelial tissue and (B) cells are modeled as (C) a network and (D) polyhedron, respectively, using the 3D vertex model. Two apical constriction modes are modeled: (E) surface contraction of the ring, and (F) line contraction of the boundary with the adjacent cell.

The 3D vertex model represented the shape of a cell as a polyhedron consisting of vertices and edges (Honda *et al*. 2004; Okuda *et al*. 2013a). The tissue was represented as an aggregate of multiple cells (Fig. 1A,B), where the vertices and edges of each cell are shared with neighboring cells. The vertices and edges composed a network that represents the entire shape of the tissue (Fig. 1C,D).

The equation of motion of vertex i with position vector $x_{i}$ at time t is:

(1) $$\eta \left(\frac{dr_{i}}{dt}-V_{i}\right)=-\nabla_{i}U.$$

Here, η is the friction coefficient and $V_{i}$ is the mean velocity vector of vertex i. In the 3D vertex model, vertex i was directly connected to four adjacent vertices by edges. Indexing the directly connected vertices as j(i), the mean velocity vector can be defined as:

(2) $$V_{i}=\frac{1}{5}\left(\frac{dr_{i}}{dt}+\sum_{j(i)}\frac{dr_{j(i)}}{dt}\right).$$

The right hand side of Equation (1) represents a force exerted on vertex i derived from the total energy function U, which represents the mechanical properties and morphogenetic events of the cells as:

(3) $$U=\sum_{j^{c}}^{\mathrm{cell}}u_{j^{c}}^{v}+u_{j^{c}}^{s}+u_{j^{c}}^{h}+u_{j^{c}}^{\mathrm{ac}},$$

where $\sum_{j^{c}}^{\mathrm{cell}}$ indicates a summation over all of the cells. The energy function U includes the cell volume elastic energy $u_{j^{c}}^{v}$, cell surface elastic energy $u_{j^{c}}^{s}$, cell height elastic energy $u_{j^{c}}^{h}$, and apical constriction energy $u_{j^{c}}^{\mathrm{ac}}$. The mathematical expressions for these energy functions are defined as:

(4) $$u_{j^{c}}^{v}\left(v_{j^{c}}\right)=\frac{1}{2}k^{v}{\left(\frac{v_{j^{c}}}{v_{j^{c}}^{\mathrm{eq}}}-1\right)}^{2},$$

(5) $$u_{j^{c}}^{s}\left(s_{j^{c}}\right)=\frac{1}{2}k^{s}{\left(\frac{s_{j^{c}}}{s_{j^{c}}^{\mathrm{eq}}}-1\right)}^{2},$$

(6) $$u_{j^{c}}^{h}\left(h_{j^{c}}\right)=\frac{1}{2}k^{h}{\left(\frac{h_{j^{c}}}{h_{j^{c}}^{\mathrm{eq}}}-1\right)}^{2},$$

(7) $$u_{j^{c}}^{\mathrm{ac}}\left(p_{j^{c}}\right)=\frac{1}{2}k^{\mathrm{ac}}p_{j^{c}}^{2}\;\left(\text{surface}\;\text{contraction}\right),$$

(8) $$u_{j^{c}}^{\mathrm{ac}}\left(l_{j^{c}}^{e}\right)=\frac{1}{2}k^{\mathrm{ac}(e)}{l_{j^{c}}^{e}}^{2}\left(\text{line}\;\text{contraction}\right).$$

These mathematical expressions were derived from a 3D vertex model of epithelial cells (Okuda *et al*. 2013b; Inoue *et al*. 2016). The $j^{c}$‐th cell's volume $v_{j^{c}}$, surface area $s_{j^{c}}$, height $h_{j^{c}}$, apical circumferential length $p_{j^{c}}$, and edge length $l_{j^{c}}^{e}$ between the $j^{c}$‐th cell and its adjacent cells are represented as variables. The superscript eq in several variables in Equations (4), (5), (6) indicates the value in the stress‐free state. The constants $k^{v}$, $k^{s}$, $k^{h}$, and $k^{\mathrm{ac}}$ ($k^{\mathrm{ac}(e)}$) are the volume elasticity, surface elasticity, height elasticity, and apical constriction constant of the cell, respectively.

Equation (7) indicates that the entire perimeter of the apical surface contracts, while Equation (8) indicates that only the boundary line with a specific cell contracts. These represent the two apical constriction modes. Equation (7) captures the surface contraction mode, and Equation (8) captures the line contraction mode (Fig. 1E,F). To examine how a particular apical constriction mode affects epithelial invagination, Equation (7) was employed in *Mechanical role in apical constriction and cell proliferation* and *Role of the spatial pattern of contractile cells in invagination*, and Equation (8) was used in *Mode of apical constriction in invagination*.

Equations (7) and (8) are quadratic functions of variables $p_{j^{c}}$ and $l_{j^{c}}^{e}$, respectively, and do not include the linear terms that would express a line tension (Fletcher *et al*. 2014). However, since the linear terms only change the net natural length that gives the minimum energy value with respect to apical constriction, for simplicity we do not consider them here.

All model constants are listed in Table 1. Here, to focus on invagination caused by the spatial pattern of the contractile cells, we ignored the asymmetry of physical properties that depend on epithelial polarity except for the contractile cells, and assumed a flat homogeneous epithelial monolayer sheet. To solve Equation (1), the parameter values were normalized by unit length ${\left(v^{\mathrm{eq}}\right)}^{1/3}$, unit time $\eta {\left(v^{\mathrm{eq}}\right)}^{2/3}/k_{B}T$, and unit energy $k_{B}T$. Here, $k_{B}T$ is formally introduced to normalize the energy parameters. To express cell proliferation, we employed a polyhedron‐division model and its parameters for the 3D vertex model (Okuda *et al*. 2013c), where we adopted a cell cycle time with mean $\tau_{\mathrm{cc}}\;=\;100$ and standard deviation 0.1*τ*_cc_.

**Table 1:** Model parameters

Symbol	Value	Descriptions
Physical parameters of the energy functions		
$k^{v}$	80	Volume elasticity of Equation (4)
$k^{s}$	1.0	Area elasticity of Equation (5)
$k^{h}$	4.0 × 10^−2^	Height elasticity of elongating cell of Equation (6)
$k^{\mathrm{ac}}$	$4.0\;\times \;10^{-4}$	Apical constriction constant (surface contraction) of Equation (7)
$k^{\mathrm{ac}(e)}$	$4.0\;\times \;10^{-3}$	Apical constriction constant (line contraction) of Equation (8)
$v^{\mathrm{eq}}$	1.0	Cell volume in stress‐free state of Equation (4)
$s^{\mathrm{eq}}$	$2v^{\mathrm{eq}}/h^{\mathrm{eq}}+2\sqrt{2\sqrt{3}v^{\mathrm{eq}}h^{\mathrm{eq}}}$	Cell surface area (hexagonal prism) in stress‐free state of Equation (5)
$h^{\mathrm{eq}}$	1.0	Cell height in stress‐free state of Equation (6)
Numerical parameters for computational simulations		
η	1.0	Friction coefficient of vertex of Equation (1)
Δt	$1.0\;\times \;10^{-6}$	Time step size for numerical integration of Equation (1)
$\Delta t_{r}$	$1.0\;\times \;10^{-3}$	Time interval at which the network reconnection rule is attempted
$\Delta l_{\mathrm{th}}$	$1.0\;\times \;10^{-2}$	Threshold edge length, below which a local network is reconnected

### Simulation conditions

**Figure 2:**
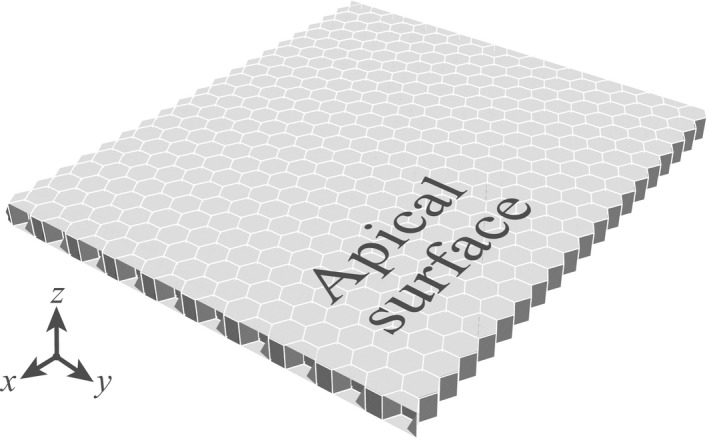
A flat monolayer is employed as an initial condition. The apical surface is defined on the positive z‐axis, and the basal surface on the negative.

The initial shape of the tissue was a flat epithelial monolayer sheet as shown in Figure 2. There were 400 hexagonal columnar cells in the calculation area. The polarity of the epithelial tissue in the initial state was assumed to coincide with the z‐axis. Periodic boundaries were employed for boundary conditions.

### Tissue deformation

The magnitude of the tissue deformation at time t, $m^{d}\left(t\right)$, was defined as the absolute value of the difference between the maximum and minimum values of the *z*‐component of the centroid position vector, $g^{c}$, of each cell in the tissue.

(9) $$m^{d}\left(t\right)=\max Z_{c}\left(t\right)-\min Z_{c}\left(t\right).$$

Here, $Z_{c}\left(t\right)$ is the set of z‐components of the centroid position of each cell at time t.

To quantify the central position of the deformation, we defined the deformation region as the set $P^{d}\left(t\right)$ as:

(10) $$P^{d}\left(t\right)=\left\{g^{c}\left(t\right)|\left(g^{c}\left(t\right)\cdot e_{z}-z_{\mathrm{ave}}^{g^{c}}\left(t\right)\right)>z_{\mathrm{sd}}^{g^{c}}\left(t\right)\right\}.$$

Then, the central position of the deformation, $\bar{p^{d}}\left(t\right)$, at time t was defined as the arithmetic mean of the element $g^{c}\left(t\right)$ of the set $P^{d}\left(t\right)$. Here, the set $P^{d}\left(t\right)$ is a set of ones in which the deviation of the *z*‐component at the centroid position of the cell exceeds the standard deviation, $z_{\mathrm{sd}}^{g^{c}}\left(t\right)$. The vector $e_{z}$ is a unit vector in the *z*‐direction, and $z_{\mathrm{ave}}^{g^{c}}\left(t\right)$ is obtained by averaging the *z*‐component of the centroid position in all the cells.

The depth of the invagination was quantified by Bessel functions that represent solutions of the Helmholtz equation in cylindrical coordinates (i.e., the radial component of the out‐of‐plane deformation of a circular elastic sheet). We measured the displacement in the z‐direction with respect to the radial axis, r, and expressed displacements u(t,r) using a superposition of Bessel functions by the least squares method (Arfken *et al*. 2012). Then, we obtained the displacement, $u_{o}\left(t\right):=u\left(t,0\right)$, at the origin as the depth of the invagination at time t.

## Results

### Mechanical role in apical constriction and cell proliferation

**Figure 3:**
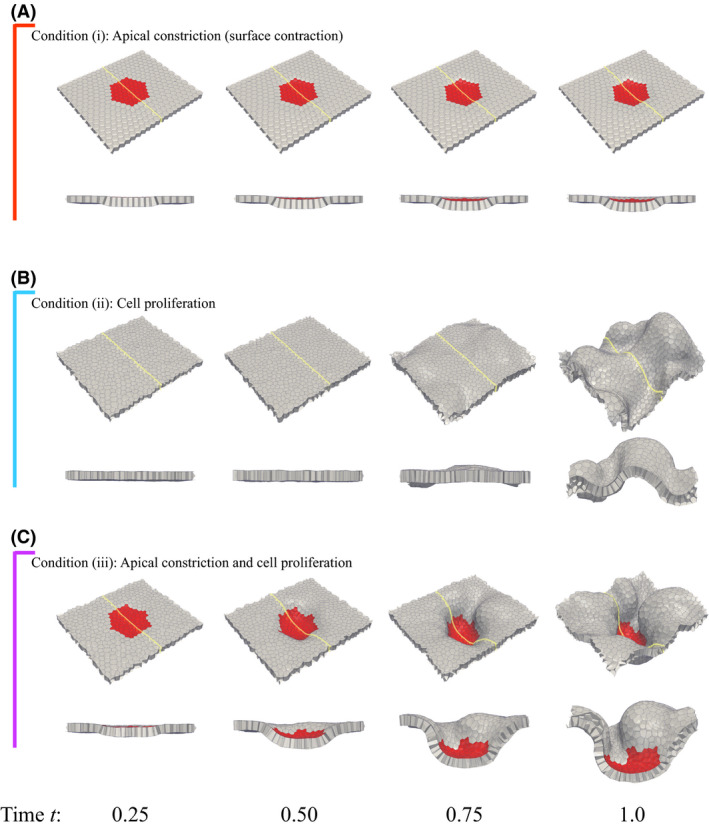
Snapshots over time t (cell cycle) of tissue deformations simulated under conditions of (A) apical constriction (condition i), (B) cell proliferation (condition ii), and (C) both apical constriction and cell proliferation (condition iii). The yellow line represents the line of the cross‐section shown below each figure. The spatial pattern in red indicates the placement of the contractile cells by apical constriction for (A, C). Cell proliferation occurs in the whole model for (B, C). The apical side is the visible side of the figure.

We examined tissue deformation as a function of (i) apical constriction, (ii) cell proliferation, and (iii) both apical constriction and cell proliferation. A circular pattern was used for the spatial pattern of the contractile cells under conditions (i) and (iii). Here, a regular hexagonal lattice represented the circular pattern, with each side initially consisting of nine cells. Cell proliferation occurred in whole for conditions (ii) and (iii). Simulation results are shown in Figure 3. Conditions (i), (ii), and (iii) resulted in a shallow basally‐convex invagination of the pattern (Fig. 3A), a large undulation of the tissue (Fig. 3B), and a large basally‐convex invagination of the pattern (Fig. 3C), respectively. Figure 3B shows that the tissue is shaped like a wave. This is due to buckling as a result of cell proliferation under periodic boundary conditions, because tissue deformation was limited to conform to the periodic boundaries. As will be shown below, such deformation occurs in random places.

**Figure 4:**
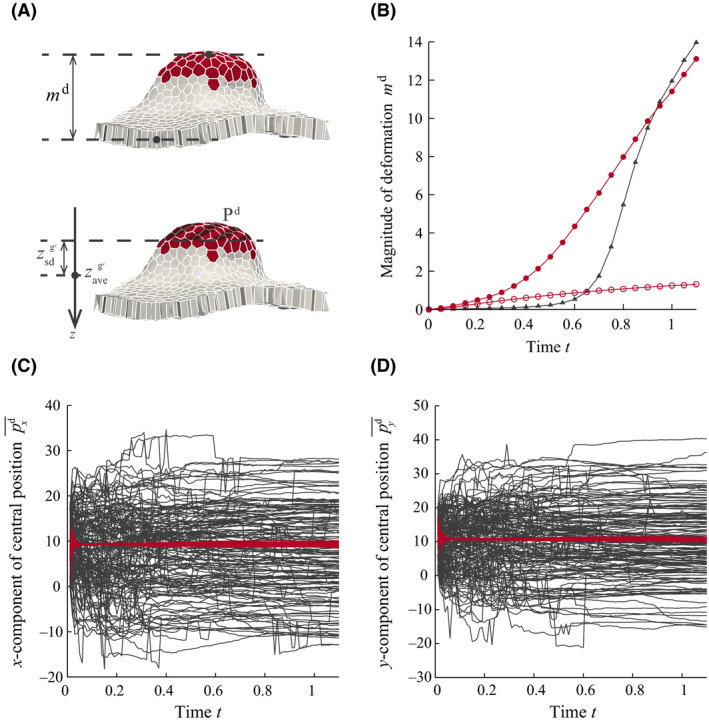
(A) Schematic definition of the magnitude of the tissue deformation, $m^{d}$ and deformation region (hatched line), $P^{d}$. (B) The magnitude of the tissue deformation presented in Figure 3 (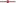 (i) Apical constriction; 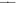 (ii) Cell proliferation; 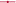 (iii) Apical constriction and cell proliferation). (C, D) The central position $\left(\bar{p_{x}^{d}},\bar{p_{y}^{d}}\right)$ of the deformation with respect to time for each sample (*N* = 150) in conditions ii (black) and iii (red) (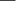 (ii) Cell proliferation; 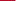 (iii) Apical constriction and cell proliferation). The central position in condition ii appears random for each sample. The central position in condition iii coincides with the centroid of the spatial pattern of the contractile cells, (x,y)=(10,10), for each sample. Time t is indicated in cell cycle units.

The magnitude and location of the deformation along the z‐direction was quantified (Fig. 4A) by the method described in *Tissue deformation*. The magnitude of the deformation, $m^{d}\left(t\right)$, for conditions (i)–(iii) is shown in Figure 4B. Conditions (ii) and (iii), in which cell proliferation occurred, resulted in a larger deformation compared with condition (i), in which cell proliferation did not occur. Comparing conditions (ii) and (iii), apical constriction resulted in an earlier deformation. Because apical constriction slightly bent the sheet to be basally convex as shown in Figure 3A, tissue deformation originated in this region and expanded using elastic energy stored in the tissue from cell proliferation. Consequently, the buckling of the epithelial cell sheet due to cell proliferation switched to invagination. Therefore, the mechanical roles of cell proliferation and apical constriction were the amplification of the tissue deformation and determination of the location of the tissue deformation, respectively.

We further examined the central position of the deformation, $\bar{p^{d}}$, for 150 samples with different random number seeds. Figure 4(C,D) shows the central position of the deformation with respect to time for each sample, which typically coincided with the centroid of the spatial pattern with apical constriction, while it was random in the absence of apical constriction, confirming that the spatial pattern of the contractile cells determined the location of the invagination.

### Role of the spatial pattern of contractile cells in invagination

**Figure 5:**
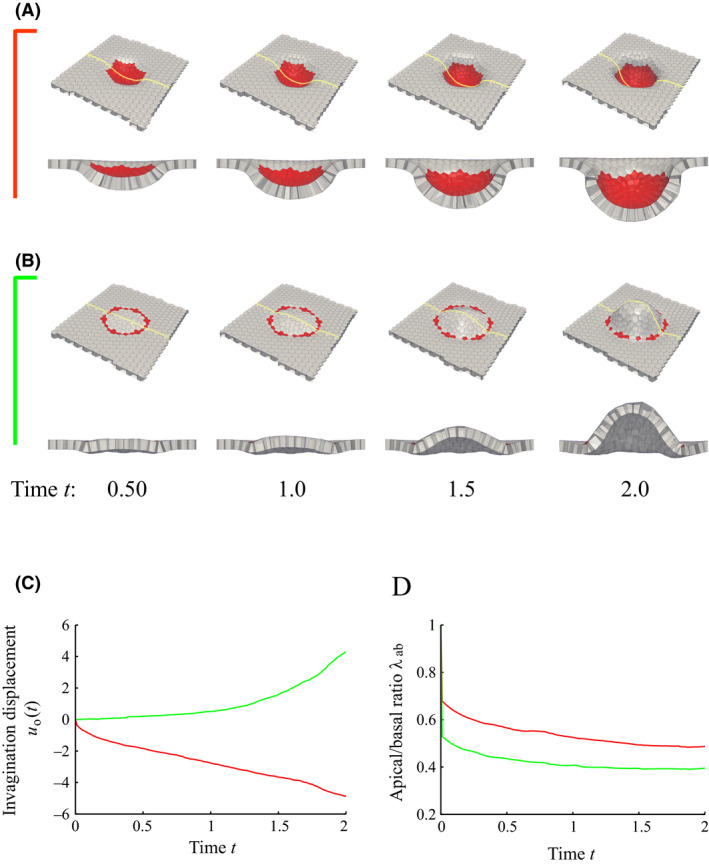
Snapshots over time t (cell cycle) of tissue invagination when the spatial pattern of the contractile cells by apical constriction (red) is (A) circular and (B) circumferential. The apical side is the visible side in the figure. The yellow line represents the line of the cross‐section shown below each figure. (C) The initial central point of the pattern is taken as the origin of the cylindrical coordinates, and the time evolution of the displacement in the z‐direction of the origin, $u_{o}\left(t\right)$, is shown (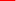 Circular pattern; 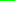 Circumference pattern). (D) The apical to basal area ratio, $\lambda_{\mathrm{ab}}$, of the contractile cell as a function of time t.

We next determined how differences in the spatial pattern of the contractile cells participating in apical constriction affected invagination. We considered (i) circular and (ii) circumferential spatial patterns (Fig. 5A,B). To use the same cell proliferation condition in both patterns, the area in which the cell proliferation occurs was the same in both patterns. Therefore, the only difference between the two conditions was whether the pattern topologically contained a hole or not. This topology difference caused a large difference in invagination; therefore it has important implications in the deformation of epithelial tissue. Figure 5(A–C) shows that the directions of invagination were opposite. A basally convex shape emerged from the circular pattern, while an apically convex shape emerged from the circumferential pattern. Because the epithelium sheet has an apico‐basal polarity, the direction of invagination is very important for establishing organs. This result indicated that the tissue could be deformed to either direction solely as a function of the spatial pattern of the contractile cells. What remains unclear is exactly how the spatial pattern determines the invagination direction.

**Figure 6:**
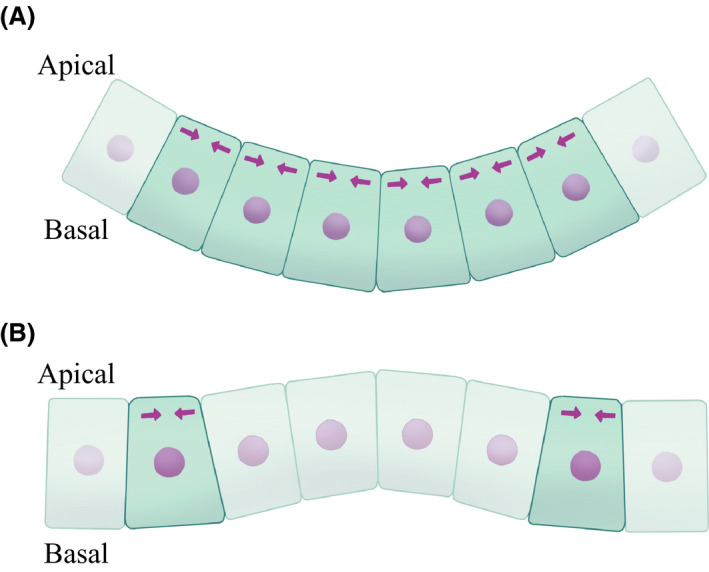
Schematic representation of cell shapes in the apico‐basal axis under (A) circular pattern and (B) circumferential pattern. In both patterns, contractile cells become apically narrow wedge‐like shapes by apical constriction (indicated by the arrow). (A) In the case of the circular pattern, the apical area is smaller than the basal area since the wedge shapes are arranged side by side, which produces the basal convex shape. (B) In the case of the circumferential pattern, the normal direction of the contact surface is inclined toward the apical direction since the wedge‐shaped cells surround the non‐contractile cells. Therefore, the inside of the circle becomes apically convex.

To investigate the differences in cell shapes between the two patterns, the shape of the contractile cells was quantified by the ratio of apical to basal area, $\lambda_{\mathrm{ab}}$, and is shown in Figure 5D. Under both conditions, $\lambda_{\mathrm{ab}}$ decreased with time, indicating that the cells changed to an apically‐narrow wedge shape. Therefore, on the cellular scale, almost the same shape was obtained from either pattern. However, since the arrangement of such wedge‐shaped cells is different for the two patterns, we considered the relationship between the arrangement of the cells and the direction of the invagination. These cell shapes restrict how the surface of the tissue on the apical side connects. Because wedge‐like cells are located next to each other in the circular pattern, the tissue surface area on the apical side is smaller than on the basal side, causing the tissue shape to become basally convex (Fig. 6A). However, wedge‐like cells are not located next to each other along the radial axis in the circumferential pattern, while non‐contractile cells are located next to each other. The normal direction of the contact surface between the contractile and non‐contractile cells inside the pattern was inclined in the apical direction so as to conform to the shape of the contractile cell. Because the reaction force due to the cell proliferation occurred along this normal direction, the tissue inside the pattern showed an apically convex invagination (Fig. 6B).

### Mode of apical constriction in invagination

In the previous section, we used surface contraction by apical constriction, in which the apical ring squeezed the apical surface of the cell. It is known, however, that there is another mode of apical constriction termed line contraction, which may occur locally on an edge between adjacent cells. In this section, we examine how differences in the apical constriction mode affect tissue invagination even when the topology of the spatial pattern of the contractile cells is equivalent. For the simulations, the circumferential pattern was used, and a contractile force was generated at the boundary line in contact with the cell inside the circumferential pattern. Cell proliferation occurred in the region enclosed by the circumferential pattern.

**Figure 7:**
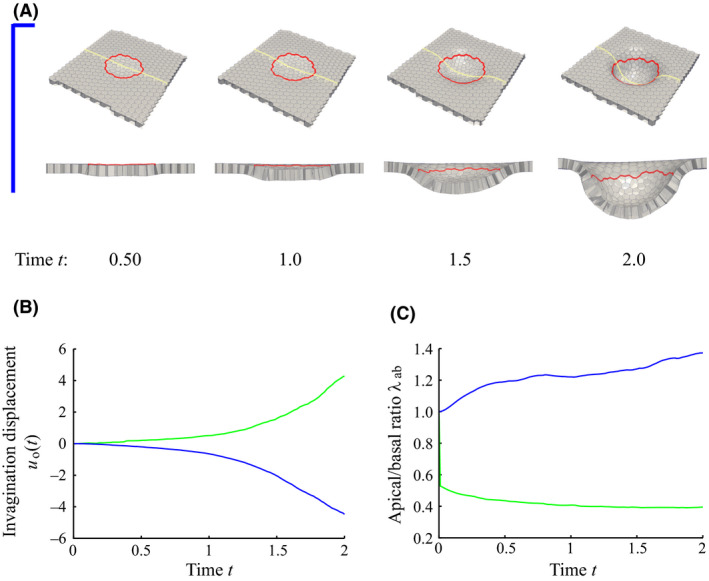
Snapshots over time t (cell cycle) of tissue invagination based on a circumferential pattern of contractile cells. (A) Apical constriction acts only on the boundary line that is in contact with the cell inside the circumferential pattern (line contraction). The apical side is the visible side in the figure. The yellow line represents the line of the cross‐section shown below each figure. (B) The initial central point of the pattern is taken as the origin of the cylindrical coordinates, and the time evolution of the displacement in the z‐direction of the origin, $u_{0}\left(t\right)$, is shown (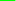 Surface contraction; 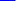 Line contraction). (C) The apical to basal area ratio, $\lambda_{\mathrm{ab}}$, of the contractile cell as a function of time t. For (B) and (C), data labeled with surface contraction are the same as that with circumferential pattern in Figure 5.

Figure 7(A,B) shows that the contractile line along the circumferential pattern produced a basally convex invagination, while surface contraction in the same pattern produced an apically convex invagination as shown in Figure 5(B). Therefore, the directions of invagination are opposite.

**Figure 8:**
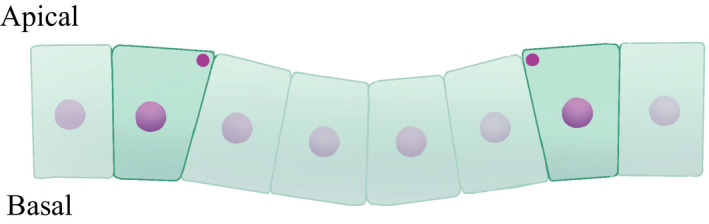
Schematic representation of the cell shapes in the apico‐basal axis under the condition of the circumferential pattern with line contraction (the dot indicates the contractile line in the cross‐section). Because centripetal motion is forced on arbitrary points on the circumference only on the apical side when the circumference shrinks, contractile cells become basally narrow wedge‐like shapes. Therefore, the tissue surrounded by the circumferential pattern becomes a basally convex shape.

The shape of the contractile cells was quantified by the ratio of apical to basal area, $\lambda_{\mathrm{ab}}$, and is shown in Figure 7(C). $\lambda_{\mathrm{ab}}$ increased with time in the case of the line contraction, indicating that the cells became basally narrow wedge‐like shapes; this is because centripetal motion is forced on arbitrary points on the circumference only on the apical side when the circumference shrinks. The normal direction of the bounding contact surface between the contractile and inner cells across the circumference was inclined in the basal direction (Fig. 8), which is opposite of the surface contraction mode. Quantitatively, we estimated the inclination angle of the contact surface between the contractile cell and the inner cell by measuring the cell height and apical and basal widths when the tissue invagination seemed to start (at t=1.0 cell cycle). The contact surface was inclined toward the apical side by about 12° in the case of surface contraction, while it was inclined toward the basal side by about 5° in the case of line contraction. Therefore, the reaction force due to the cell proliferation occurred along this normal direction, resulting in an apically (Fig. 6B) and a basally convex (Fig. 8) invagination in the case of surface or line contraction, respectively. Thus, even if the spatial pattern is the same, there is a possibility that the direction of invagination may change because of a different mode of apical constriction.

## Discussion

In this study, we examined the mechanical roles of cell proliferation and apical constriction, and determined that they are responsible for expanding the degree of tissue deformation and determining the location of the deformation, respectively. We showed that the direction of invagination depended on the spatial pattern of the contractile cells. In addition, the direction of invagination differed between surface and line contraction modes of apical constriction, even when the spatial pattern was the same. The invagination direction can be understood from the alignment of wedge‐shaped cells.

From a physical point of view, surface contraction by apical constriction can also be expressed by surface energy proportional to apical area. By performing simulations of invagination using a surface energy function, we confirmed that the choice of energy function expressing the surface contraction did not affect the directionality of the invagination (Appendix I). This is because the surface energy function can be converted mathematically to the form of Equation (7) under the assumption that the apical surface is a regular hexagon. From a mechanical point of view, because buckling of tissue depends on tissue size, we examined whether the system size affected the directionality, and qualitatively confirmed that the result did not change (Appendix II).

Basal convex invagination was observed in the *Drosophila* spiracle (Simões *et al*. 2006), in which an enrichment of Myosin II and activated Rho 1 at the apical side occurred in a circular pattern. Basal convex invagination was also observed in the *Drosophila* tracheal placode, in which a circumferential pattern with line contraction was seen via accumulation of Myosin at the boundary of cells with high and low EGFR activity (Nishimura *et al*. 2007). During the optic cup formation, apical constriction in the hinge area along the circumference of the tissue and apically convex invagination has been observed (Eiraku *et al*. 2011). Our simulation results are consistent with these experimental results.

In our simulations, the cell division axis was assumed to follow the long axis of the cell (Hertwig's rule); therefore the tissue growth was isotropic. However, the mitotic axis may be controlled by the morphogen concentration gradient, for example, in the *Drosophila* wing imaginal disc, the mitotic spindle tends to be oriented along the proximal‐distal axis (Mao *et al*. 2011). The determination of the direction of invagination will not be affected by the orientation of the cell division axis if the cell division generates no bending moment in the tissue. Because the dividing nucleus is known to translocate apically (Ragkousi & Gibson 2014), the biased location of the nucleus may cause the generation of a bending moment in the tissue, possibly affecting the invagination direction.

From the mechanical point of view, because invagination due to cell proliferation can be interpreted as a type of sheet buckling due to the increase of in‐plane elastic stress, the direction of invagination is indeterminate. To determine the direction of the invagination, mechanical asymmetry can be required along the apical‐basal polarity. One possible source of asymmetry is an apically narrow wedge‐like cell shape induced by apical constriction as shown in this study. Another possible source of asymmetry is a difference in the mechanical properties between the apical and the basal sides of the epithelium. In addition, the surrounding mechanical properties are important for both the invagination direction and the tissue shape after invagination. In experiments on 3D tissue formation from Madin‐Darby canine kidney (MDCK) cells using an *in vitro* system, it was observed that the height of the protrusion structure (tulip‐hat‐like shape) decreased as the viscosity of the substrate increased (Imai *et al*. 2015). Investigation of the cell division orientation axis and the surrounding mechanical properties are future research directions.

A basal constriction induced the morphogenesis of a tulip‐hat‐like shape in the MDCK cell sheet (Imai *et al*. 2015). Although we did not incorporate basal constriction in this study, a comparison can be made between the *in vitro* experiments and our simulation results from a mechanical point of view. With active force generation, the tulip‐hat‐like shape protruded to the opposite side of the contractile side. Additionally, phosphorylated myosin regulatory light chains were observed on the outermost edge of the MDCK cell sheet, indicating a circumferential pattern. According to our simulation results, the invagination that satisfies these geometric conditions is the circumferential pattern with line contraction, which coincides with the experimental results.

Other mechanical effects can be attributed to the surroundings. When an invagination of bottle cells occurs during gastrulation in *Xenopus* embryos, their apical surfaces shrink mainly along the animal‐vegetal direction (Keller *et al*. 2003). However, bottle cells in isolated explants without adjacent tissues show an isotropic contraction and become circular apices. This experiment suggests that even if the intrinsic contraction is isotropic, an anisotropic deformation may occur because of mechanical influences from surrounding tissues. Throughout our study we assumed that all cells have the same physical properties and used a periodic boundary condition to enforce isotropy, therefore anisotropic deformation did not occur. To reproduce the actual *in vivo* phenomena, it is necessary to incorporate differences in boundary conditions and cell physical properties from tissue to tissue. How anisotropic deformation emerges from an isotropic spatial pattern can then by analyzed.

Finally, we would like to point out that actively contracting the cell surface through apical or basal constrictions is not the only way to trigger invagination. If the cell sheet is adhered to the matrix or another tissue and the elastic moduli of the surroundings are high enough, it is expected that cell height elongation would result in an invagination to the matrix side (Clausi & Brodland 1993; Inoue *et al*. 2016). For example, if an incompressible basement membrane is formed, cell elongation causes preferential reduction in the apical area of the cell to maintain the cell volume, and thus, the cell becomes an apically narrow wedge‐like shape, causing invagination. Furthermore, from the physical point of view, it is suggested that when the cell sheet proliferates, a folding pattern is generated because of the difference in growth rate between the cell sheet and the matrix (Hannezo *et al*. 2011; Amar & Jia 2013; Tallinen *et al*. 2016). Such a folding pattern is of interest for understanding intestinal villi and the wrinkle structure of the brain.

## Funding

Y. I. acknowledges support from MEXT KAKENHI (15H05861, 15H05856). Y. I. and T. A. acknowledge support from the Platform Project for Supporting in Drug Discovery and Life Science Research (Platform for Dynamic Approaches to Living System) from the Japan Agency for Medical Research and Development (AMED).

## Figures and Tables

**Figure A1:**
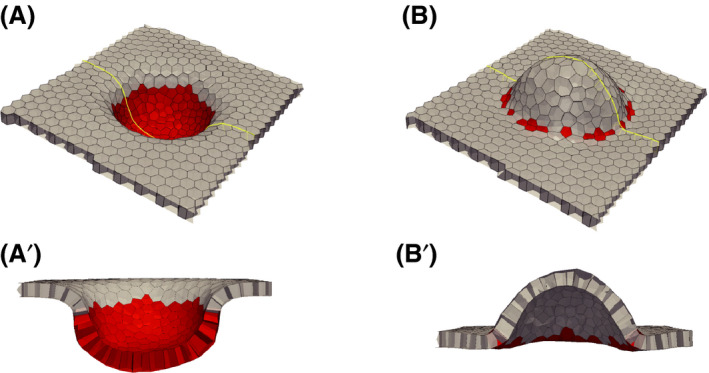
Snapshots at time t=2.0 (cell cycle) of tissue invagination when the spatial pattern of the contractile cells by apical constriction (red) is (A) circular and (B) circumferential. The apical side is the visible side in the figure. The yellow line represents the line of the cross‐section shown in (A’) and (B’). Surface contraction is expressed by surface energy, Equation (11), instead of Equation (7).

**Figure A2:**
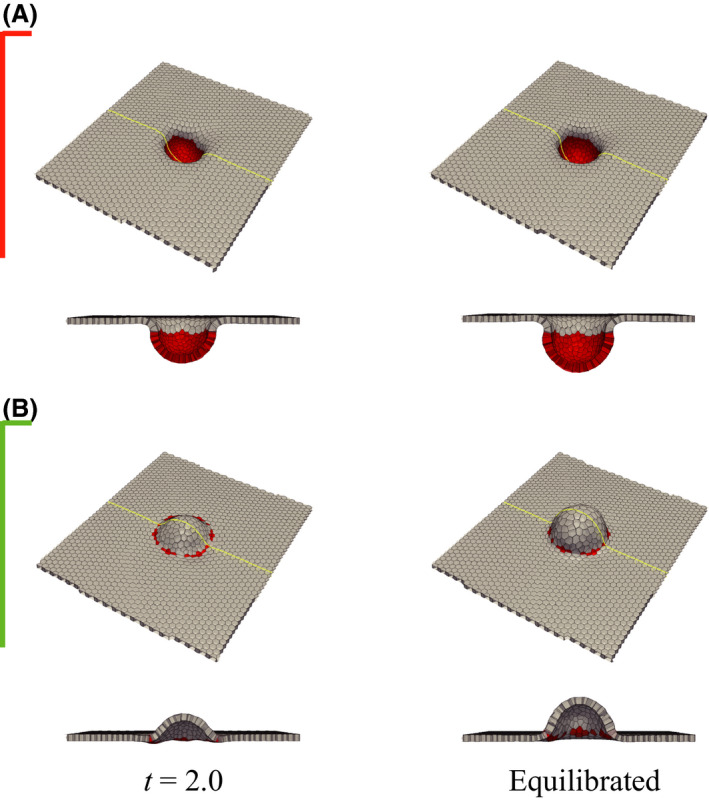
Snapshots at time t=2.0 (cell cycle) of tissue invagination and equilibrated tissue shapes when the spatial pattern of the contractile cells by apical constriction (red) is (A) circular and (B) circumferential. The apical side is the visible side in the figure. The yellow line represents the line of the cross‐section shown below each figure. The tissue with double width in both the *x*‐ and *y*‐axes of that in Figure 2 is employed. The equilibration of tissue shape is performed undergoing apical constriction, in which cell proliferation ceased after t=2.0 (cell cycle).

## Appendix I Energy function of surface contraction

Equation (7) expresses surface contraction by apical constriction. This energy function does not include the apical area, $a_{j^{c}}$, as a variable directly. Therefore, we investigated whether the invagination direction changed when we employed a surface energy function. The surface energy is expressed by the product of the apical surface tension, σ, and apical area, $a_{j^{c}}$ as follows:

(11)
$$u_{j^{c}}^{\mathrm{ac}}\left(a_{j^{c}}\right)=\sigma a_{j^{c}}.$$

We used Equation (11) instead of Equation (7), and performed simulations of tissue invagination with circular and circumferential patterns where $\sigma \;=\;2.8\times 10^{-3}$($∼4\sqrt{3}k^{\mathrm{ac}}$). Figure A1 shows snapshots of tissue invagination at t=2.0 (cell cycle), which coincided fairly well with the results shown in Figure 5. Thus, our conclusions do not change if either form of the energy function expressing surface contraction is used. This is because we can rewrite Equation (7) as Equation (11) with $\sigma \;=\;4\sqrt{3}k^{\mathrm{ac}}$ if we assume that the apical surface shape is approximated by a regular hexagon. If the apical surface shape deforms to the extent that it cannot be approximated as a regular planar shape, this assumption will be violated. The lumen side of a tubular structure with a small radius is one possible shape that may violate this assumption.

## Appendix II Effect of system size in invagination

Our simulations showed that the invagination of the tissue was localized by the patterned tension. However, because the critical buckling load depends on the size of tissue, we investigated whether the system size would affect the deformation of the rest of the tissue. We performed simulations using a double‐width tissue of that in Figure 2. Figure A2 shows that slight bending deformation was observed around the pattern; however, the invagination was essentially localized in the patterned region. We also examined an equilibrating simulation of tissue deformation undergoing apical constriction, in which cell proliferation ceased after t=2.0 (cell cycle). After an additional 1‐cell‐cycle equilibration, the tissue shape was nearly relaxed. Although the invagination was deep in both patterns, the equilibrated shape was not significantly different from the shape before equilibration.
